# Patterning in Birthweight in India: Analysis of Maternal Recall and Health Card Data

**DOI:** 10.1371/journal.pone.0011424

**Published:** 2010-07-02

**Authors:** Malavika A. Subramanyam, Leland K. Ackerson, S. V. Subramanian

**Affiliations:** 1 Center for Integrative Approaches to Health Disparities, School of Public Health, University of Michigan, Ann Arbor, Michigan, United States of America; 2 Department of Community Health and Sustainability, University of Massachusetts Lowell, Lowell, Massachusetts, United States of America; 3 Department of Society, Human Development and Health, Harvard School of Public Health, Boston, Massachusetts, United States of America; UCL Institute of Child Health, United Kingdom

## Abstract

**Background:**

National data on birthweight from birth certificates or medical records are not available in India. The third Indian National Family Health Survey included data on birthweight of children obtained from health cards and maternal recall. This study aims to describe the population that these data represent and compares the birthweight obtained from health cards with maternal recall data in terms of its socioeconomic patterning and as a risk factor for childhood growth failure.

**Methodology/Principal Findings:**

The analytic sample consisted of children aged 0 to 59 months with birthweight data obtained from health cards (n = 3227) and maternal recall (n = 16787). The difference between the card sample and the maternal recall sample in the distribution across household wealth, parental education, caste, religion, gender, and urban residence was compared using multilevel models. We also assessed the ability of birthweight to predict growth failure in infancy and childhood in the two groups. The survey contains birthweight data from a majority of household wealth categories (>5% in every category for recall), both genders, all age groups, all caste groups, all religion groups, and urban and rural dwellers. However, children from the lowest quintile of household wealth were under-represented (4.73% in card and 8.62% in recall samples). Comparison of data across health cards and maternal recall revealed similar social patterning of low birthweight and ability of birthweight to predict growth failure later in life. Children were less likely to be born with low birthweight if they had mothers with over 12 years of education compared to 1–5 years of education with relative risk (RR) of 0.79 (95% confidence interval [CI]: 0.52, 1.2) in the card sample and 0.70 (95% CI: 0.59, 0.84) in the recall sample. A 100 gram difference in a child's birthweight was associated with a decreased likelihood of underweight in both the card (RR: 0.95; 95% CI: 0.94, 0.96) and recall (RR: 0.96; 95% CI: 0.96, 0.97) samples.

**Conclusions:**

Our results suggest that in the absence of other sources, the data on birthweight in the third Indian National Family Health Survey is valuable for epidemiologic research.

## Introduction

Birthweight is a key indicator of the health trajectory of a child. In addition to being an intrinsic endpoint[1], low birthweight is associated with increased risk of numerous adverse health outcomes in childhood[2], and adulthood[3]. Even though India is reported to have one of the highest rates of low birthweight in the world[4], the ideal source of birthweight data in the form of birth certificates or hospital discharge data does not exist in India at the national level. The National Family Health Survey in its third round (NFHS-3), the equivalent of the Demographic and Health Surveys (DHS) in India, obtained information on the birthweight of the children based on maternal recollection as well as by asking mothers to show a health card that records the child birthweight in cases where the delivery was institutional. While there are legitimate concerns related to birthweight data from the DHS, especially the extent of missingness and its representativeness[5], in the sheer absence of any alternative source of birthweight data for research as well as surveillance it is important to investigate the utility of the NFHS-3 data on birthweight. In this study, we investigate the extent and characteristics of the sub-population to which the birthweight data from NFHS-3 can be generalized. Doing so allows researchers to use these data with a clear picture of the population they represent. Since the birthweight data were obtained from two sources in the NFHS-3 (maternal recall and health cards), we sought to determine what populations the data from the two groups represent. We also investigated the extent of similarity in socioeconomic patterning of birthweight in the two groups, and assessed the ability of birthweight from each of these sources to predict subsequent growth failure in childhood.

## Methods

### Data source

We analyzed data from the third round of the National Family Health Survey of India (NFHS-3) conducted in 2005–06. The NFHS was established to generate representative data at the national and state levels on population and health indicators, with special emphasis on maternal and child health outcomes. The NFHS is the Indian equivalent of the DHS assessments which use standard model questionnaires widely used in more than 80 developing countries[6]. The target population for the 2005–2006 NFHS was children aged 0 to 59 months, women aged 15 to 49 years, and men aged 15 to 54 years[7].

### Sampling Plan

Multistage stratified sampling methods were used to create a sample representing individuals from all 29 Indian states[8], [9]. The sample size of each state was proportional to the population and the sample was stratified by urban and rural residence within states. Within state strata, primary sampling units (PSUs) were selected based on probability proportional to population and were villages or clusters of villages in rural areas, and census enumeration blocks within urban areas. Households were randomly selected within PSUs. Within selected households, all women aged 15 to 49 years were eligible to be respondents in the survey. Through a two-phase data collection process, 12 states were surveyed between November 2005 and May 2006, and the remaining 17 states were surveyed between April and August 2006[8]. From 116 652 selected households, 124 385 women participated in the survey out of 131 596 who were eligible, yielding an overall response rate of 94.5%, which ranged between 90% and 99% among all states[9]. Of the 124 385 women who participated in the survey, 36 850 reported having 1 or more live births less than five years before completing the survey. These women reported 51 555 live births within this time frame, and we restricted the sample for this study to singletons alive at the time of survey (n = 48 065). Of this sample, 28 051 children were missing data on birthweight. The sample for the health card analyses comprised of all 3227 children that had their birthweight recorded on a health card, while the sample for the maternal recall analysis comprised of all 16 787 children that had their birthweight recorded based on maternal self-report (Table 1).

**Table 1:** Weighted frequency of children across household, parent and child covariates by method of birthweight report.

Characteristics		Birthweight from card		Birthweight from recall		Missing data on birthweight		Total
		N	Weighted%	N	Weighted%	N	Weighted%	
Total		3227	100	16787	100	28049	100	48063
**Household covariates**								
Wealth (quintile)*	First (highest)	1504	40.32	6537	30.66	2255	5.29	10296
	Second	967	28.80	4847	27.13	5052	13.88	10866
	Third	476	17.58	2941	19.60	6584	20.40	10001
	Fourth	199	8.58	1590	14.00	7014	27.47	8803
	Fifth	81	4.73	872	8.62	7144	32.96	8097
Caste*	Scheduled caste	379	13.88	2577	17.79	5540	22.43	8496
	Scheduled tribe	434	4.95	1966	6.12	5396	11.29	7796
	Other backward class	1015	39.91	5250	38.07	9531	42.08	15796
	General class	1244	37.28	6335	35.40	6312	20.83	13891
	No caste	155	3.98	659	2.63	1270	3.38	2084
Religion*	Hindu	2109	76.20	12153	80.14	18753	77.33	33015
	Muslim	536	15.74	2158	13.41	5332	19.00	8026
	Christian	444	5.38	1576	2.70	2765	1.43	4785
	Sikh	35	1.03	362	1.86	427	1.03	824
	Other	103	1.65	538	1.89	772	1.21	1413
Urban residence*	City	1358	36.65	5943	27.26	3892	8.53	11193
	Town	636	14.98	3454	16.76	3090	6.89	7180
	Village	1233	48.37	7390	55.98	21067	84.59	29690
**Parent covariates**								
Maternal education*	Zero	347	12.88	2798	21.45	16092	64.78	19237
(Years of schooling)	1 to 5	301	9.80	2145	13.97	4479	14.38	6925
	6 to 12	1939	58.82	9151	52.59	7040	19.87	18130
	>12	640	18.50	2693	11.99	437	0.96	3770
	Missing	0		0		1	0	1
Paternal education*	Zero	241	8.65	1748	13.43	9336	37.4	11325
(Years of schooling)	1 to 5	309	9.47	1906	12.59	4702	16.06	6917
	6 to 12	1937	60.42	9256	54.23	12104	40.09	23297
	13 to 15	480	13.54	2662	13.56	1157	3.82	4299
	>15	241	7.39	1079	5.50	356	1.24	1676
	Missing	19	0.53	136	0.70	394	1.40	549
**Child covariates**								
Age in months*	Infant	838	26.10	3382	20.33	5216	18.80	9436
	13 to 23	701	20.89	3449	20.89	5315	19.10	9465
	24 to 35	646	19.95	3440	20.18	5511	19.44	9597
	36 to 47	538	16.69	3290	19.63	6010	21.10	9838
	48 to 59	504	16.37	3226	18.97	5997	21.56	9727
Gender*	Female	1530	46.51	7834	46.12	13707	48.59	23071
	Male	1697	53.49	8953	53.88	14342	51.41	24992

*P value of chi square test of association between source of data (card and recall) and social factors was <0.001 for household wealth, religion, urban residence, maternal education, paternal education and age. For caste p = 0.009 and for gender p = 0.76.

### Outcomes

Birthweight was measured in grams (whether from health card or maternal recall) and was used as a continuous variable. We also created a binary indicator of low birthweight based on whether the birthweight was less than 2500 grams or not. We assessed the ability of the birthweight data to predict the child's growth failure as measured by underweight, severe underweight, stunting, severe stunting, wasting and severe wasting. We used data on anthropometry available in the NFHS, which applied the World Health organization (WHO) standards to compute the Z scores for weight-for-age, height-for-age, and weight-for-height. Following WHO guidelines, children with a Z score below two standard deviations for weight-for-age, height-for-age, and weight-for-height were classified as underweight, stunted, and wasted respectively. Children whose Z scores were below three standard deviations were classified as having severe growth failure. Height was measured to the nearest 1 millimeter using a measuring board, taking care to measure length for children less than 2 years of age. Weight was measured using the UNICEF Uniscale to the nearest 100 grams. The data available to us were missing data on anthropometry for 8% of eligible children at the national level because these children were missing data on month and year of birth or had grossly improbable height or weight measurements[9].

### Socioeconomic Variables

We used the following variables to compare the socioeconomic patterning of birthweight and low birthweight: household wealth, maternal education, paternal education, caste, residence (urban vs. rural), gender, and religion. Household wealth was operationalized as possession of household assets. The dataset contained an index of 33 household assets and characteristics that had been created using principal components analysis (PCA)[10]. We weighted the PCA scores in the dataset by the household sampling weights to ensure that the distribution was representative of all the households in India and then divided the households into quintiles. Categorical variables for maternal and paternal education were created using data on the number of years of schooling. Maternal education had the following categories: no education (0 years of schooling), primary education (1–5 years), secondary education (6–12 years), and greater than secondary (>12 years of education). The paternal education variable had an additional category for those with greater than 15 years of education.

The caste variable was coded as scheduled caste, scheduled tribe, “other backward class”, general caste, and missing or no caste. This classification (using terminology adopted by the Government of India) focuses more on the socially disadvantaged castes, and all privileged caste groups are represented in the “general” group[11]. Urban residence was categorized as residing in cities, towns or villages. The religion of the head of the household was assigned to each individual, with indicators denoting Hindu, Muslim, Christian, Sikh, and other religions. All analyses were adjusted for the age of the child, maternal age, and birth order.

### Analysis

To describe the population that this sample of children with birthweight data represents, we first calculated the frequency distribution of children, by source of birthweight (and for those missing birthweight data), across categories of the covariates (age, gender, household wealth, maternal education, paternal education, caste, and religion). We calculated mean and standard deviation of birthweight and prevalence of low birthweight across the socioeconomic categories in datasets that compared children with data from health cards and maternal recall data. These descriptive analyses used survey analytic methods that account for clustering by primary sampling units and the sampling weights.

We then estimated multivariable regression models with birthweight and low birthweight as two separate outcomes, in order to quantify the adjusted association between the socioeconomic indicators and the outcomes. We additionally estimated models with the anthropometric measures as the outcomes, to evaluate the ability of birthweight and low birthweight in predicting subsequent growth failure in the child. In order to account for the multilevel structure of the data (children nested within households within clusters within states) and to account for clustering, we used a multilevel modeling approach, with random effects specified for households, clusters, and states. We fit linear models for birthweight and generalized linear models with a Poisson distribution and a log link function for low birthweight and anthropometric measures. A Poisson model for binary outcomes is a suitable choice in instances where the outcomes, such as here, are not rare events[12], [13]. For presentation, we report the beta estimates and standard errors for linear models and relative risk (RR) estimates and 95% confidence intervals (CI) for the Poisson models.

In order to compare birthweight data obtained from health cards with data from maternal recall, all multivariable models were fit for two sets of data: a) children with birthweight data from health cards, and b) children with birthweight data as per maternal recall. As an exploratory exercise, we reproduced all analyses in a pooled dataset which included records from the health card and maternal samples.

### Ethical Review

The 2005–06 National Family Health Survey was conducted under the scientific and administrative supervision of the International Institute for Population Sciences, (IIPS) Mumbai, India. The IIPS is a regional center for teaching, training and research in population studies, and is associated with the Ministry of Health and Family Welfare, Government of India. The institute conducted an independent ethics review of 2005–06 NFHS protocol. Data collection procedures were also approved by the ORC Macro institutional review board. The study was reviewed by Harvard School of Public Health Institutional Review Board and was considered as exempt from full review as the study was based on an anonymous public use data set with no identifiable information on the survey participants.

## Results

### Covariate distribution by source of birthweight data

The distribution of children across the social factors between the birthweight data obtained from health cards and maternal recall, while statistically significantly different, did not vary to a great extent (Table 1). The children were mostly from households in the highest wealth quintile (40.32% and 30.66%), with mothers (77.3% and 64.6%) and fathers (81.88% and 73.99%) who had greater than 6 years of education, residing in villages (48.37% and 55.98%) and representing all religion, caste, age and gender groups. Notably, there were no cells with zero frequency in the card and maternal recall datasets. In both datasets, there were more than 8% of children in the 4^th^ wealth quintile, and more than 5% in every category of maternal and paternal education. The only group that had a negligible presence was the lowest wealth quintile. Those missing data on birthweight were mostly children from the lower two quintiles of household wealth (60.5%), living in villages (84.59%), whose mothers did not go to school (64.78%).

### Descriptive statistics of birthweight sample

Mean birthweight varied by sociodemographic characteristics in both the datasets (Table 2). The birthweight of children with mothers having over 12 years of formal education (2938 grams, SD: 31.35) and fathers with over 15 years of formal education (2984 grams, SD: 49.71), were substantially higher than those whose parents had no education (2827 grams, SD: 61.18; and 2830 grams, SD: 70.62; respectively) in the card sample. The corresponding figures in the recall sample were 2924 (SD: 17.17) versus 2796 (SD: 20.98) for maternal education and 2956 (SD: 26.86) versus 2780 (SD: 23.64) for paternal education.

**Table 2:** Mean birthweight (standard deviation) and frequency of low birthweight (LBW) across covariates in the card and recall samples.

		Card			Recall		
Characteristics		N	Mean (SD)	% of low birthweight (95% CI)	N	Mean (SD)	% of low birthweight (95% CI)
Wealth (quintile)	First (highest)	1504	2932.57 (25.72)	13.72 (11.12,16.31)	6537	2878.67 (11.87)	16.26 (14.85,17.68)
	Second	967	2830.82 (27.96)	19.40 (15.86,22.93)	4847	2812.06 (14.35)	20.22 (18.53,21.90)
	Third	476	2758.05 (39.70)	21.49 (16.39,26.59)	2941	2814.59 (17.41)	22.58 (20.50,24.66)
	Fourth	199	2737.95 (49.48)	23.33 (16.40,30.26)	1590	2790.63 (25.68)	24.08 (21.17,27.00)
	Fifth	81	3001.10 (110.19)	19.86 (11.29,28.43)	872	2772.09 (28.73)	24.43 (21.43,27.43)
Caste	Scheduled caste	379	2814.40 (46.40)	20.93 (15.40,26.46)	2577	2798.44 (19.12)	21.78 (19.54,24.02)
	Scheduled tribe	434	2878.99 (94.22)	25.57 (16.71,34.43)	1966	2846.42 (32.53)	22.11 (18.71,25.52)
	Other backward class	1015	2883.38 (27.48)	15.95 (12.79,19.11)	5250	2830.78 (12.46)	20.15 (18.68,21.63)
	General class	1244	2845.10 (24.24)	17.71 (14.95,20.46)	6335	2833.51 (13.55)	19.46 (17.93,20.99)
	No caste	155	2878.97 (60.22)	17.45 (9.34,25.56)	659	2814.74 (41.85)	22.20 (17.52,26.88)
Religion	Hindu	2109	2858.18 (20.08)	17.70 (15.44,19.95)	12153	2817.07 (8.91)	20.57 (19.49,21.64)
	Muslim	536	2876.92 (41.63)	17.72 (13.07,22.38)	2158	2860.50 (22.67)	19.98 (17.53,22.43)
	Christian	444	2928.90 (52.35)	13.66 (7.58,19.74)	1576	2978.61 (41.37)	15.40 (11.33,19.46)
	Sikh	35	2830.74 (98.36)	24.86 (11.05,38.68)	362	2769.81 (40.67)	23.16 (17.95,28.37)
	Other	103	2524.24 (114.95)	34.29 (15.45,53.13)	538	2825.72 (61.20)	19.36 (12.46,26.25)
Urban	City	1358	2864.30 (29.37)	17.05 (13.83,20.28)	5943	2846.83 (13.99)	18.78 (17.11,20.45)
	Town	636	2879.34 (36.51)	13.80 (9.63,17.97)	3454	2857.90 (17.21)	17.03 (14.96,19.11)
	Village	1233	2848.97 (24.92)	19.67 (16.86,22.48)	7390	2807.26 (11.87)	22.15 (20.80,23.49)
Maternal education	Zero	347	2827.07 (61.18)	25.84 (19.50,32.18)	2798	2792.62 (20.98)	24.83 (22.67,27.00)
	1 to 5	301	2802.65 (49.80)	17.87 (12.05,23.68)	2145	2806.15 (21.76)	23.60 (21.02,26.19)
	6 to 12	1939	2850.65 (20.82)	17.17 (14.83,19.52)	9151	2823.46 (9.91)	19.29 (18.08,20.50)
	>12	640	2938.43 (31.35)	14.34 (10.63,18.05)	2693	2924.39 (17.17)	13.38 (11.33,15.42)
Paternal education	Zero	241	2830.40 (70.62)	21.08 (14.37, 27.80)	1748	2780.14 (23.64)	24.65 (21.97,27.32)
	1 to 5	309	2760.71 (47.65)	22.16 (16.03, 28.30)	1906	2830.38 (23.39)	22.01 (19.38,24.65)
	6 to 12	1937	2847.06 (23.48)	18.48 (15.89,21.06)	9256	2813.21 (10.59)	20.14 (18.88,21.41)
	13 to 15	480	2929.20 (33.35)	13.21 (9.13,17.29)	2662	2869.23 (19.11)	17.91 (15.53,20.29)
	>15	241	2983.80 (49.71)	12.32 (7.03,17.61)	1079	2955.54 (26.86)	13.61 (10.52,16.70)
	Missing	19	2936.37 (129.48)	8.80 (0.43,17.17)	136	2838.00 (138.06)	27.51 (16.74,38.28)
Age (months)	0 to 11	838	2832.62 (27.36)	19.01 (15.41, 22.60)	3382	2814.37 (15.08)	19.61 (17.76, 21.47)
	12 to 23	701	2841.23 (33.76)	19.01 (14.97, 23.06)	3449	2797.28 (17.23)	22.67 (20.67, 24.68)
	23 to 35	646	2854.91 (36.94)	18.44 (14.13, 22.75)	3440	2820.87 (16.57)	21.28 (19.38, 23.17)
	36 to 47	538	2867.60 (36.23)	15.63 (11.56, 19.71)	3290	2849.35 (15.44)	18.52 (16.71, 20.34)
	48 to 59	504	2920.80 (39.21)	15.96 (11.46, 20.46)	3226	2854.20 (17.73)	19.60 (17.62, 21.59)
Gender	Female	1530	2812.22 (20.92)	19.56 (16.90, 22.22)	7834	2777.30 (10.77)	21.72 (20.44,23.01)
	Male	1697	2899.95 (24.17)	16.33 (13.74,18.91)	8953	2868.67 (10.69)	19.22 (18.01,20.42)

There was a gradient in prevalence of low birthweight across categories of household wealth with the lowest prevalence among children from the highest quintile (13.72%; 95% CI: 11.12, 16.31), and the highest prevalence among children from the lowest quintile (19.86%; 95% CI: 11.29, 28.43) in the card sample. Similar patterns were observed in the recall sample. In both card and recall samples the disparities by parental education was similar to that observed with birthweight. Weighted prevalence of low birthweight was lower among boys (16.33%; 95% CI: 13.74, 18.91) compared to girls (19.56%; 95% CI: 16.90, 22.22) in the card sample. The gender differences in prevalence of low birthweight were similar in the recall sample as well. Residents of towns (13.80%; 95% CI: 9.63, 17.97 in the card sample and 17.03%; 95% CI: 14.96, 19.11 in the recall sample) had the lowest prevalence of low birthweight, while rural dwellers had the highest prevalence (19.67%; 95% CI: 16.86, 22.48 in the card sample and 22.15%; 95% CI: 20.80, 23.49 in the recall sample). The differentials across age and caste groups were not substantial.

### Social patterning of birthweight and low birthweight

The social patterning of birthweight among children whose mothers reported their birthweight differed from that among children in the health card sample (Table 3). Children from households in the lowest wealth quintile were not substantially different in terms of birthweight from those in the third quintile among those whose mother reported birthweight (β: −16.73; SE: 27.61). Among children whose weight was recorded from a health card, however, those from households in the lowest wealth quintile had substantially higher birthweight than those from the third quintile (β: 198.00; SE: 76.83). Children born to a scheduled tribe family were not different from those born to general caste families among those whose mother reported birthweight (β: 38.61; SE: 24.53). Among children whose weight was recorded from a health card, however, those who were born to a scheduled tribe family had substantially higher birthweight than those born to general caste families (β: 132.50; SE: 47.50).

**Table 3:** Predicted difference* in birthweight (gm) across categories of covariates by source of data.

Characteristics		Birth weight (card)		Birth weight (recall)	
Household covariates		Beta	standard error	Beta	standard error
Wealth (quintile)	First (highest)	121.10	38.39	49.03	18.88
	Second	56.77	35.08	14.89	16.49
	Third	Ref	Ref	Ref	Ref
	Fourth	−17.63	51.35	−10.51	21.14
	Fifth	198.00	76.83	−16.73	27.61
Caste	Scheduled caste	−26.66	38.60	−3.01	17.25
	Scheduled tribe	132.50	47.50	38.61	24.53
	Other backward class	14.00	28.51	2.28	14.25
	General class	Ref	Ref	Ref	Ref
	No caste	29.70	53.79	0.67	29.52
Religion	Hindu	Ref	Ref	Ref	Ref
	Muslim	42.74	34.63	58.78	18.37
	Christian	74.37	42.04	142.40	26.88
	Sikh	95.56	113.80	82.14	46.95
	Other	46.32	68.57	53.86	32.01
Urban residence	City	−26.83	31.27	−29.77	16.80
	Town	7.98	34.10	−5.70	17.46
	Village	Ref	Ref	Ref	Ref
**Parent covariates**					
Maternal education	Zero	44.03	48.59	−23.63	19.91
(Years of schooling)	1 to 5	Ref	Ref	Ref	Ref
	6 to 12	35.43	39.62	18.96	17.02
	>12	98.10	51.63	70.16	24.48
Paternal education	Zero	−43.97	46.25	−31.97	19.98
(Years of schooling)	1 to 5	−93.86	38.98	−5.89	17.66
	6 to 12	Ref	Ref	Ref	Ref
	13 to 15	31.34	34.69	34.71	16.50
	>15	54.58	47.21	59.29	24.60
	Missing	81.04	138	−30.34	57.21
**Child covariates**					
Gender	Female	−62.36	20.90	−92.43	10.12
	Male	Ref	Ref	Ref	Ref

*Models additionally adjusted for age, maternal age, and birth order.

There were no discrepancies, however, in the social patterning of low birthweight, whether birthweight data had been recorded from a health card, or obtained by maternal recall (Table 4). In the health card sample, children were less likely to be born with low birthweight if they were from the highest quintile of households compared to the middle quintile (RR: 0.69; 95% CI: 0.51, 0.92) or had mothers with over 12 years of education compared to 1–5 years of education (RR: 0.79; 95% CI: 0.52,1.2). Girls were more likely to be born with low birthweight than boys (RR: 1.17; 95% CI: 0.99, 1.38). Similar social patterning was observed in the maternal recall samples.

**Table 4:** Relative risk (95% confidence interval) of low birthweight* across covariates by source of data.

Characteristics		low birthweight (card)	low birthweight (recall)
Household covariates		RR (95% CI)	RR (95% CI)
Wealth (quintile)	First (highest)	0.69 (0.51, 0.92)	0.80 (0.71, 0.91)
	Second	0.86 (0.67, 1.11)	0.90 (0.81, 1.00)
	Third	1.00 [Reference]	1.00 [Reference]
	Fourth	1.00 (0.70, 1.43)	0.98 (0.86, 1.12)
	Fifth	0.93 (0.54, 1.61)	0.99 (0.84, 1.17)
Caste	Scheduled caste	1.10 (0.83, 1.45)	1.04 (0.93, 1.16)
	Scheduled tribe	0.89 (0.61, 1.29)	0.95 (0.81, 1.12)
	Other backward class	0.89 (0.71, 1.12)	1.02 (0.93, 1.11)
	General class	1.00 [Reference]	1.00 [Reference]
	No caste	0.98 (0.64, 1.52)	1.02 (0.84, 1.25)
Religion	Hindu	1.00 [Reference]	1.00 [Reference]
	Muslim	1.05 (0.81, 1.37)	0.98 (0.88, 1.10)
	Christian	0.74 (0.50, 1.07)	0.74 (0.61, 0.90)
	Sikh	0.93 (0.44, 1.98)	0.90 (0.68, 1.19)
	Other	0.80 (0.44, 1.43)	0.78 (0.61, 0.99)
Urban residence	City	0.96 (0.76, 1.20)	1.05 (0.95, 1.16)
	Town	0.90 (0.69, 1.17)	0.94 (0.84, 1.04)
	Village	1.00 [Reference]	1.00 [Reference]
**Parent covariates**			
Maternal education	Zero	1.04 (0.73, 1.46)	1.03 (0.91, 1.16)
(Years of schooling)	1 to 5	1.00 [Reference]	1.00 [Reference]
	6 to 12	0.88 (0.66, 1.18)	0.83 (0.75, 0.93)
	>12	0.79 (0.52, 1.20)	0.70 (0.59, 0.84)
Paternal education	Zero	0.93 (0.66, 1.32)	1.04 (0.92, 1.18)
(Years of schooling)	1 to 5	1.03 (0.78, 1.38)	1.05 (0.94, 1.17)
	6 to 12	1.00 [Reference]	1.00 [Reference]
	13 to 15	0.86 (0.64, 1.17)	0.91 (0.81, 1.03)
	>15	0.86 (0.56, 1.32)	0.85 (0.70, 1.03)
	Missing	0.72 (0.18, 2.98)	1.38 (0.99, 1.92)
**Child covariates**			
	Female	1.17 (0.99, 1.38)	1.13 (1.06, 1.22)
	Male	1.00 [Reference]	1.00 [Reference]

*Models additionally adjusted for age, maternal age and birth order, and conditional on random effect.

### Birthweight and childhood growth failure

We observed statistically significant associations between both birthweight (in units of 100 grams) and low birthweight and growth failure later in life (Table 5). A 100 gram difference in a child's birthweight in the card sample was associated with decreased likelihood of stunting (RR: 0.97; 95% CI: 0.96, 0.98), underweight (RR: 0.95; 95% CI: 0.94, 0.96), and wasting (RR: 0.96; 95% CI: 0.94, 0.97) at an older age in the card sample. The corresponding figures in the recall sample were 0.98 (95% CI: 0.97, 0.98), 0.96 (95% CI: 0.96, 0.97), and 0.97 (95% CI: 0.96, 0.97) in the recall sample. In the card sample, low birthweight was associated with increased likelihood of stunting (RR: 1.41; 95% CI: 1.18, 1.67), underweight (RR: 1.77; 95% CI: 1.48, 2.12), and wasting (RR: 1.67; 95% CI: 1.32, 2.11) in later life. A similar pattern was seen in the recall sample for stunting (RR: 1.32; 95% CI: 1.24, 1.41), underweight (RR: 1.57; 95% CI: 1.46, 1.68) and wasting (RR: 1.41; 95% CI: 1.28, 1.55).

**Table 5:** Relative risk (95% CI) for the association between growth failure* and birthweight/low birthweight by source of data.

		Card	Recall
Outcome	Predictor	RR (95% CI)	RR (95% CI)
Stunting	Birthweight	0.97 (0.96, 0.98)	0.98 (0.97, 0.98)
	low birthweight	1.41 (1.18, 1.67)	1.32 (1.24, 1.41)
Underweight	Birthweight	0.95 (0.94, 0.96)	0.96 (0.96, 0.97)
	low birthweight	1.77 (1.48, 2.12)	1.57 (1.46, 1.68)
Wasting	Birthweight	0.96 (0.94, 0.97)	0.97 (0.96, 0.97)
	low birthweight	1.67 (1.32, 2.11)	1.41 (1.28, 1.55)
Severe stunting	Birthweight	0.97 (0.95, 0.99)	0.97 (0.96, 0.98)
	low birthweight	1.46 (1.09, 1.95)	1.50 (1.35, 1.66)
Severe underweight	Birthweight	0.93 (0.91, 0.95)	0.97 (0.96, 0.98)
	low birthweight	2.94 (2.12, 4.09)	1.80 (1.59, 2.04)
Severe wasting	Birthweight	0.96 (0.93, 0.99)	0.96 (0.95, 0.97)
	low birthweight	1.62 (1.09, 2.43)	1.41 (1.19, 1.67)

*Birthweight and low birthweight were included in separate models. The RRs for birthweight are for 100 gm difference. All models additionally adjust for household wealth, caste, religion, urban residence, maternal and paternal education, age, gender, maternal age, and birth order and are conditional on random effects.

The mean birthweight and proportion of low birthweight across covariates (Table S1), socioeconomic patterning of low birthweight (Table S3) and the association between birthweight and growth failure at a later age (Table S4) in the pooled sample were similar to the results in both the card and recall samples. The socioeconomic disparities in birthweight (Table S2) were closer to the results from the recall sample but not the card sample.

## Discussion

This study has two key findings. First, we found that contrary to prevalent beliefs [5], [14] the Indian National Family Health Survey-3 contains birthweight data from a majority of household wealth categories, both genders, all age groups, all caste groups, all religion groups and urban and rural dwellers. Second, our comparison of low birthweight data across two sources—health card records and maternal recall—revealed that the data were similar with regards to their socioeconomic patterning, and especially in predicting growth failure in childhood using low birthweight.

While there are multiple studies of birthweight in India[15], [16], [17], [18], [19], [20], [21], [22], [23], [24], [25], [26], [27], [28], [29], some of them well designed community-based cohorts[30], [31], [32], [33], they remain local studies. The NFHS-3 is the only source that provides data from a sample of individuals across the country and includes data from a majority of sociodemographic groups of the Indian population. The households in the poorest wealth quintile, however, were much underrepresented. We found that there was some representation of every level of the sociodemographic factors we examined in both of the sources of birth weight data: the health cards and maternal recall. However, the proportion of missingness was not the same across these social factors. As might be expected, there was relatively greater missing data among the poorest, least educated, those belonging to disadvantaged caste groups, and rural dwellers. Despite this social patterning in missingness, these findings highlight the sub-population of India to whom analyses of birthweight are generalizable: children across India, of both genders, aged less than five years, of all caste and religion groups, and belonging to middle and wealthier households. Arguably this encompasses a large proportion of the population of children under age five in India.

Although we posit that these data represent a specific subgroup of children within the Indian context, it may be possible to extrapolate these data to the larger population. For example, researchers interested in using these data might be able to come up with weighting schemes to account for the social patterning in missingness. Such weighting techniques have already been proposed to account for the heaping of birthweight recall data at multiples of 500 grams in order to estimate the prevalence of low birthweight at the country level[5].

Our comparison of birthweight from two sources of the data in the survey, health cards and maternal recall, revealed that the differences between the two might not be as large as has previously been discussed, especially for low birthweight[5]. This method of comparison, wherein the measures of birthweight in two groups are compared on their associations with other variables, with prior knowledge as to what these associations look like, is called “criterion related validation”[34]. Specifically, we found, in both groups, socioeconomic advantages to be associated with lower likelihood of low birthweight, consistent with previous studies[19], [21], [31]. We also found associations of birthweight and low birthweight with later growth failure that were consistent with those of earlier research in both groups[35]. These results are generalizable only to the sub-population described previously.

To our knowledge only one published study has evaluated birthweight data from the Indian NFHS[5]. This study used data pooled from 62 countries including Indian data from 1992 and 1999, but not from 2005–2006. Since we use only 2005–2006 Indian data, our study is not strictly comparable with the Blanc and Wardlaw study. While Blanc and Wardlaw address heaping in actual birthweight data, they mostly focus on an assessment of “baby size” by mothers (“larger than average”, “smaller than average”, etc). This variable provides important information that can supplement the use of actual birthweight. Dharmalingam and colleagues have suggested that this is a useful proxy for birthweight[14], citing the findings by Blanc and Wardlaw as evidence to support their use of this variable. However Blanc and Wardlaw found that, while consistent at the aggregate level, the correlation between baby size and birthweight was variable at the individual level. We posit that ignoring the data on actual birthweight in the NFHS is to ignore valuable data. Information on baby size can potentially be used in conjunction with data on birthweight to provide a more comprehensive picture.

Our results must be interpreted keeping in mind several limitations regarding the quality of the data. One assumption we have made is that birthweight data taken from the health card are reliable. Specifically, these cards are filled out by medical professionals at health care institutions while attending the birth of the child. Since these cards are typically filled out at the same time as a standard medical record by the same individual, there is little reason to think that the quality of the data from these cards would vary widely from that of other medical records, which are taken as a gold standard for medical information retrieval. Another possible limitation is that mothers in India may recall birthweight differently for malnourished children compared to those who are not malnourished. The fact that we found birthweight reported by maternal recall to have similar patterns compared with objectively measured birthweight from the health cards provides evidence that this limitation cannot completely account for our results. As the data on all children of one mother were collected at one time point, there is a chance that mothers providing data on more than one child might report similar or even identical birthweight for all their children. However, our modeling techniques account for clustering of data and moreover, we found that our findings were robust even when we dropped the records of siblings with identical birthweight. Most importantly, the purpose of our analysis is to encourage the efficient use of existing rich data. However, the extent of missing data, especially among the disadvantaged sections of the Indian population, cannot be addressed by modeling or weighting techniques. A conscientious attempt at gathering data from all socioeconomic groups would allow researchers and policy-makers to work towards prevention of low birthweight as well as reduction of social disparities in child health.

### Conclusion

Birthweight is a key variable for measuring the quality of the prenatal medical and social environment as well as predicting future individual health outcomes. Our study describes the population to which results of studies using existing birthweight data might be generalized, which is valuable in the Indian context where ensuring completion of data in a national survey is a daunting challenge, especially given the high proportion of home births. These birthweight data appear especially suitable for studies focused on examining the effect of low birthweight on other social and health outcomes. This study indicates that birthweight data from health cards and maternal recall data from the 2005–2006 NFHS can be used to study the epidemiology of low birthweight in India with appropriate cautions.

## Supporting Information

Table S1 Mean birthweight (standard deviation) and frequency of lowbirthweight (LBW) across covariates in the pooled sample (card + recall).
(0.08 MB DOC)

Table S2 Predicted difference* in birthweight (gm) across categories of covariates in the pooled sample (card + recall). Footnote: Models additionally adjusted for age, maternal age, and birth order.
(0.06 MB DOC)

Table S3 Relative risk (95% confidence interval) of low birthweight* across covariates in the pooled sample (card + recall). Footnote: *Models additionally adjusted for age, maternal age and birth order, and conditional on random effects.
(0.06 MB DOC)

Table S4 Relative risk (95% CI) for the association between growth failure* and birthweight/low birthweight in the pooled sample (card + recall). Footnote: Birthweight and low birthweight were included in separate models. The RRs for birthweight are for 100 gm difference. All models additionally adjusted for household wealth, caste, religion, urban residence, maternal and paternal education, age, gender, maternal age, and birth order and are conditional on random effects.
(0.05 MB DOC)
