# Supplementary material for: Patterning in Birthweight in India: Analysis of Maternal Recall and Health Card Data
Source: PLoS One. 2010 Jul 2;5(7):e11424. doi: 10.1371/journal.pone.0011424 (PMC2896401; doi:10.1371/journal.pone.0011424)
Supplement: Table S1 — Mean birthweight (standard deviation) and frequency of lowbirthweight (LBW) across covariates in the pooled sample (card + recall). (0.08 MB DOC) [file pone.0011424.s001.doc]

Table S1. Mean birthweight (standard deviation) and frequency of low birthweight (LBW) across covariates in the pooled sample (card + recall)

| **Characteristics** |  | **N** | **Mean (SD)** | **% of low birthweight** **(95% CI)** |
| --- | --- | --- | --- | --- |
| **Household covariates** |  |  |  |  |
| Wealth (quintile) | First (highest) | 8041 | 2888.46 (10.81) | 15.81 (14.56, 17.06) |
|  | Second | 5814 | 2814.91 (12.83) | 20.10 (18.57, 21.62) |
|  | Third | 3417 | 2807.16 (15.95) | 22.44 (20.52, 24.37) |
|  | Fourth | 1789 | 2785.7 (23.78) | 24.02 (21.28, 26.76) |
|  | Fifth | 953 | 2791.51 (28.61) | 24.05 (21.22, 26.88) |
| Caste | Scheduled caste | 2956 | 2800.31 (17.88) | 21.69 (19.58, 23.80) |
|  | Scheduled tribe | 2400 | 2850.33 (31.62) | 22.53 (19.27, 25.80) |
|  | Other backward class | 6265 | 2838.70 (11.40) | 19.53 (18.18, 20.87) |
|  | General class | 7579 | 2835.26 (12.05) | 19.20 (17.82, 20.58) |
|  | No caste | 814 | 2827.82 (36.50) | 21.24 (17.17, 25.31) |
| Religion | Hindu | 14262 | 2822.76 (8.23) | 20.17 (19.19, 21.16) |
|  | Muslim | 2694 | 2863.22 (19.50) | 19.61 (17.44, 21.78) |
|  | Christian | 2020 | 2966.09 (33.45) | 14.96 (11.57, 18.36) |
|  | Sikh | 397 | 2775.02 (38.87) | 23.31 (18.32, 28.30) |
|  | Other | 641 | 2787.03 (57.54) | 21.28 (14.71, 27.85) |
| Urban residence | City | 7301 | 2850.06 (12.70) | 18.47 (16.99, 19.94) |
|  | Town | 4090 | 2860.72 (15.57) | 16.61 (14.70, 18.53) |
|  | Village | 8623 | 2812.57 (10.81) | 21.84 (20.61, 23.07) |
| **Parent covariates** |  |  |  |  |
| Maternal education | Zero | 3145 | 2795.80 (19.90) | 24.93 (22.90, 26.97) |
| (Years of schooling) | 1 to 5 | 2446 | 2805.78 (20.00) | 23.00 (20.61, 25.40) |
|  | 6 to 12 | 11090 | 2827.79 (9.02) | 18.96 (17.86, 20.06) |
|  | >12 | 3333 | 2927.30 (14.87) | 13.58 (11.78, 15.38) |
| Paternal education | Zero | 1989 | 2785.07 (22.64) | 24.30 (21.79, 26.81) |
| (Years of schooling) | 1 to 5 | 2215 | 2822.53 (21.66) | 22.03 (19.57, 24.50) |
|  | 6 to 12 | 11193 | 2818.58 (9.70) | 19.89 (18.73, 21.04) |
|  | 13 to 15 | 3142 | 2877.88 (17.03) | 17.24 (15.11, 19.37) |
|  | >15 | 1320 | 2960.77 (24.35) | 13.38 (10.74, 16.02) |
|  | Missing | 155 | 2849.17 (123.38) | 25.39 (15.48, 35.31) |
| **Child covariate** |  |  |  |  |
| Gender | Female | 9364 | 2782.38 (9.76) | 21.41 (20.24, 22.59) |
|  | Male | 10650 | 2873.16 (9.78) | 18.81 (17.70, 19.91) |
