# Supplementary material for: Patterning in Birthweight in India: Analysis of Maternal Recall and Health Card Data
Source: PLoS One. 2010 Jul 2;5(7):e11424. doi: 10.1371/journal.pone.0011424 (PMC2896401; doi:10.1371/journal.pone.0011424)
Supplement: Table S2 — Predicted difference* in birthweight (gm) across categories of covariates in the pooled sample (card + recall). Footnote: Models additionally adjusted for age, maternal age, and birth order. (0.06 MB DOC) [file pone.0011424.s002.doc]

Table S2. Predicted difference***** in birthweight (gm) across categories of covariates in the pooled sample (card + recall)

| **Characteristics** |  |  | |
| --- | --- | --- | --- |
| **Household covariates** | | **Beta** | **standard error** |
| Wealth (quintile) | First (highest) | 62.05 | 17.03 |
|  | Second | 20.72 | 14.97 |
|  | Third | Ref | Ref |
|  | Fourth | -10.44 | 19.52 |
|  | Fifth | 3.57 | 25.87 |
| Caste | Scheduled caste | -5.1 | 15.81 |
|  | Scheduled tribe | 43.82 | 22.2 |
|  | Other backward class | 4.47 | 12.88 |
|  | General class | Ref | Ref |
|  | No caste | 1.43 | 26.19 |
| Religion | Hindu | Ref | Ref |
|  | Muslim | 60.24 | 16.52 |
|  | Christian | 121.6 | 23.39 |
|  | Sikh | 96.4 | 44.01 |
|  | Other | 50.74 | 29.16 |
| Urban residence | City | -28.92 | 15.28 |
|  | Town | -8.17 | 16.01 |
|  | Village | Ref | Ref |
| **Parent covariates** |  |  |  |
| Maternal education | Zero | -15.48 | 18.38 |
| (Years of schooling) | 1 to 5 | Ref | Ref |
|  | 6 to 12 | 21.62 | 15.62 |
|  | >12 | 74.85 | 22.08 |
| Paternal education | Zero | -35.67 | 18.33 |
| (Years of schooling) | 1 to 5 | -17.73 | 16.08 |
|  | 6 to 12 | Ref | Ref |
|  | 13 to 15 | 34.2 | 14.92 |
|  | >15 | 59.78 | 21.94 |
|  | Missing | -19.6 | 52.67 |
| **Child covariates** |  |  |  |
| Gender | Female | -88.8 | 9.13 |
|  | Male | Ref | Ref |

*Models additionally adjusted for age, maternal age, and birth order.
