# Supplementary material for: Patterning in Birthweight in India: Analysis of Maternal Recall and Health Card Data
Source: PLoS One. 2010 Jul 2;5(7):e11424. doi: 10.1371/journal.pone.0011424 (PMC2896401; doi:10.1371/journal.pone.0011424)
Supplement: Table S3 — Relative risk (95% confidence interval) of low birthweight* across covariates in the pooled sample (card + recall). Footnote: *Models additionally adjusted for age, maternal age and birth order, and conditional on random effects. (0.06 MB DOC) [file pone.0011424.s003.doc]

Table S3. Relative risk (95% confidence interval) of low birthweight* across covariates in the pooled sample (card + recall)

| **Household covariates** | | **RR (95% CI)** |
| --- | --- | --- |
| Wealth (quintile) | First (highest) | 0.78 (0.69, 0.87) |
|  | Second | 0.89 (0.81, 0.98) |
|  | Third | 1 [Reference] |
|  | Fourth | 0.98 (0.87, 1.11) |
|  | Fifth | 0.98 (0.84, 1.15) |
| Caste | Scheduled caste | 1.05 (0.95, 1.16) |
|  | Scheduled tribe | 0.97 (0.83, 1.12) |
|  | Other backward class | 1 (0.92, 1.09) |
|  | General class | 1 [Reference] |
|  | No caste | 1.02 (0.85, 1.23) |
| Religion | Hindu | 1 [Reference] |
|  | Muslim | 0.99 (0.89, 1.1) |
|  | Christian | 0.76 (0.64, 0.91) |
|  | Sikh | 0.86 (0.66, 1.13) |
|  | Other | 0.78 (0.62, 0.98) |
| Urban residence | City | 1.03 (0.94, 1.13) |
|  | Town | 0.93 (0.84, 1.03) |
|  | Village | 1 [Reference] |
| **Parent covariates** |  |  |
| Maternal education | Zero | 1.02 (0.91, 1.15) |
| (Years of schooling) | 1 to 5 | 1 [Reference] |
|  | 6 to 12 | 0.84 (0.76, 0.93) |
|  | >12 | 0.71 (0.61, 0.84) |
| Paternal education | Zero | 1.03 (0.91, 1.15) |
| (Years of schooling) | 1 to 5 | 1.05 (0.94, 1.16) |
|  | 6 to 12 | 1 [Reference] |
|  | 13 to 15 | 0.9 (0.81, 1.01) |
|  | >15 | 0.85 (0.72, 1.02) |
|  | Missing | 1.32 (0.96, 1.81) |
| **Child covariates** |  |  |
|  | Female | 1.14 (1.07, 1.22) |
|  | Male | 1 [Reference] |

*Models additionally adjusted for age, maternal age and birth order, and conditional on random effects
