# Supplementary material for: Patterning in Birthweight in India: Analysis of Maternal Recall and Health Card Data
Source: PLoS One. 2010 Jul 2;5(7):e11424. doi: 10.1371/journal.pone.0011424 (PMC2896401; doi:10.1371/journal.pone.0011424)
Supplement: Table S4 — Relative risk (95% CI) for the association between growth failure* and birthweight/low birthweight in the pooled sample (card + recall). Footnote: Birthweight and low birthweight were included in separate models. The RRs for birthweight are for 100 gm difference. All models additionally adjusted for household wealth, caste, religion, urban residence, maternal and paternal education, age, gender, maternal age, and birth order and are conditional on random effects. (0.05 MB DOC) [file pone.0011424.s004.doc]

Table S4. Relative risk (95% CI) for the association between growth failure* and birthweight/ low birthweight in the pooled sample (card + recall)

| **Outcome** | **Predictor** | **RR (95% CI)** |
| --- | --- | --- |
|  |  |  |
| Stunting | Birthweight | 0.98 (0.97, 0.98) |
|  | low birthweight | 1.33 (1.26, 1.42) |
|  |  |  |
| Underweight | Birthweight | 0.96 (0.96, 0.97) |
|  | low birthweight | 1.59 (1.49, 1.70) |
|  |  |  |
| Wasting | Birthweight | 0.97 (0.96, 0.97) |
|  | low birthweight | 1.45 (1.32, 1.58) |
|  |  |  |
| Severe stunting | Birthweight | 0.97 (0.96, 0.98) |
|  | low birthweight | 1.49 (1.36, 1.64) |
|  |  |  |
| Severe underweight | Birthweight | 0.96 (0.95, 0.96) |
|  | low birthweight | 1.90 (1.69, 2.13) |
|  |  |  |
| Severe wasting | Birthweight | 0.97 (0.96, 0.98) |
|  | low birthweight | 1.44 (1.23, 1.68) |

*Birthweight and low birthweight were included in separate models. The RRs for birthweight are for 100gm difference. All models additionally adjust for household wealth, caste, religion, urban residence, maternal and paternal education, age, gender, maternal age, and birth order and are conditional on random effects.
